# Insights into the regulatory role of Plexin D1 signalling in cardiovascular development and diseases

**DOI:** 10.1111/jcmm.16509

**Published:** 2021-04-09

**Authors:** Yi‐Fei Zhang, Yu Zhang, Dong‐Dong Jia, Hong‐Yu Yang, Meng‐Die Cheng, Wen‐Xiu Zhu, Hui Xin, Pei‐Feng Li, Yin‐Feng Zhang

**Affiliations:** ^1^ Institute for Translational Medicine The Affiliated Hospital of Qingdao University College of Medicine Qingdao University Qingdao China; ^2^ Department of Cardiology The Affiliated Hospital of Qingdao University Qingdao China

**Keywords:** angiogenesis, cardiovascular, development, disease, Plexin D1, signalling

## Abstract

Plexin D1 (PLXND1), which was previously thought to mediate semaphorin signalling, belongs to the Plexin family of transmembrane proteins. PLXND1 cooperates mostly with the coreceptor neuropilin and participates in many aspects of axonal guidance. PLXND1 can also act as both a tumour promoter and a tumour suppressor. Emerging evidence suggests that mutations in PLXND1 or Semaphorin 3E, the canonical ligand of PLXND1, can lead to serious cardiovascular diseases, such as congenital heart defects, CHARGE syndrome and systemic sclerosis. Upon ligand binding, PLXND1 can act as a GTPase‐activating protein (GAP) and modulate integrin‐mediated cell adhesion, cytoskeletal dynamics and cell migration. These effects may play regulatory roles in the development of the cardiovascular system and disease. The cardiovascular effects of PLXND1 signalling have gradually been elucidated. PLXND1 was recently shown to detect physical forces and translate them into intracellular biochemical signals in the context of atherosclerosis. Therefore, the role of PLXND1 in cardiovascular development and diseases is gaining research interest because of its potential as a biomarker and therapeutic target. In this review, we describe the cardiac effects, vascular effects and possible molecular mechanisms of PLXND1 signalling.

## 1 INTRODUCTION

Plexins (PLXNs) were discovered by Takagi and colleagues in 1987 in the *Xenopus laevis* tadpole model^1^ while the researchers were screening molecules in the optic tectum using monoclonal antibodies (MAbs). The plexin protein is an antigen of a MAb, that is, MAb‐B2, and constituted a new type one membrane glycoprotein.^2^ The new protein was subsequently renamed plexin because it preferentially binds the retinal plexiform layer.^3^, ^4^ The first human PLXNs were identified by Maestrini and colleagues in 1996,^5^ and in 1999, Tamagnone and colleagues identified 4 classes of PLXNs in vertebrates (A to D) based on the sequence similarities of their ectodomains.^6^

**FIGURE 1:**
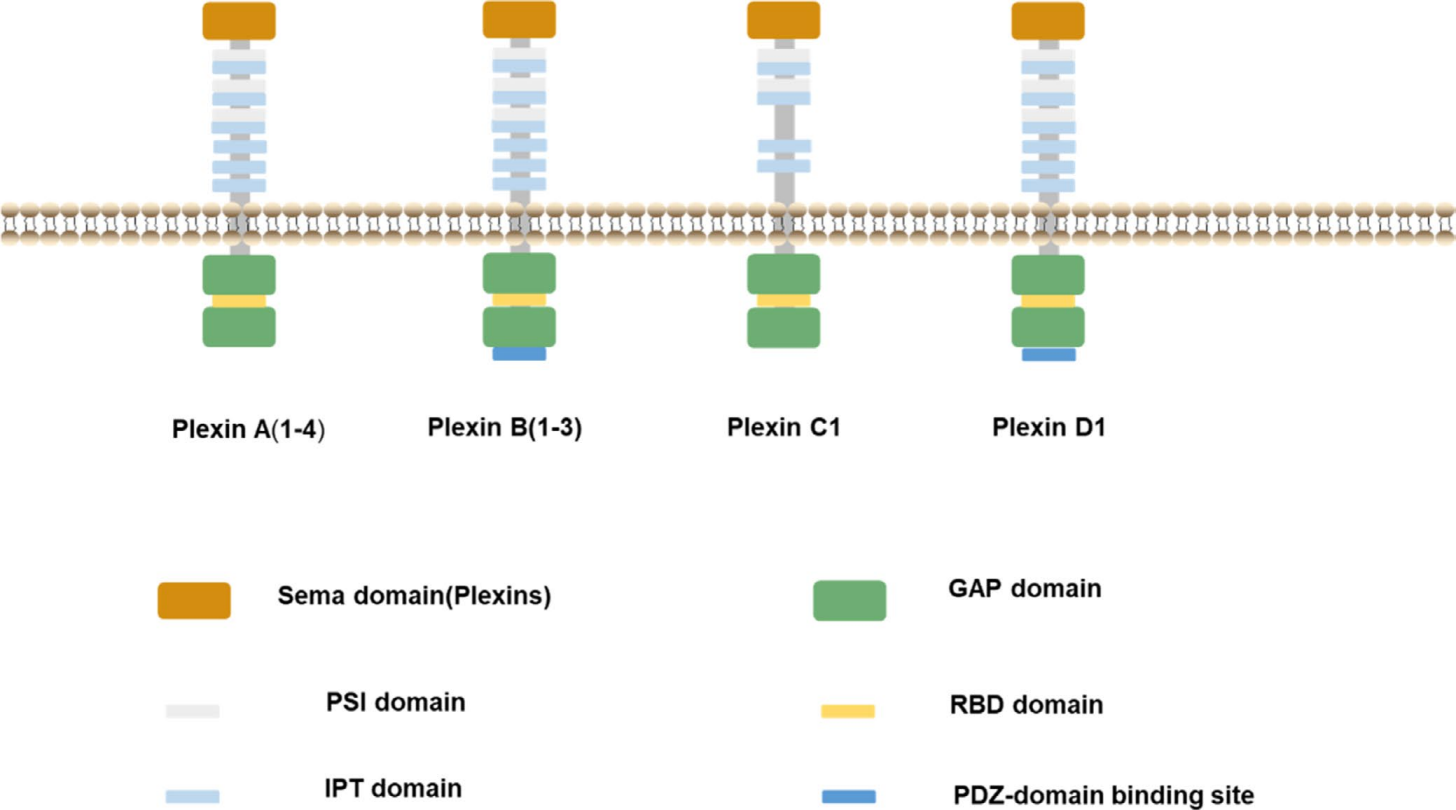
The structure of Plexins. The extracellular domains of plexins are composed of an N‐terminal sema domain, followed by three plexin‐semaphorin‐integrin (PSI) domains and six immunoglobulin domains shared by plexins and transcription factors (IPT). The cytoplasmic domain of plexins consists of a GTPase‐activating protein (GAP) domain that is separated by a Rho GTPase binding domain (RBD). One PSI and two IPT domains of Plexin C1 are absent. Plexin B1 and Plexin D1 have a COOH‐terminal PSD‐95/Dlg/ZO‐1 (PDZ) binding motif

Subsequently, studies by several research groups unravelled the structure of PLXNs (Figure 1).^7^ PLXNs contain an extracellular sema domain, one membrane‐spanning region and a cytoplasmic domain that has a GTPase‐activating protein (GAP) domain and a Rho GTPase binding domain (RBD) insert.^7^, ^8^ Although this domain is divided into two segments by the RBD in its sequence, it interweaves in three dimensions to form a typical GAP domain.^9^ The N‐terminal sema domain of the extracellular domain is followed by three plexin‐semaphorin‐integrin (PSI) domains and six immunoglobulin domains shared by plexins and transcription factors (IPT domains), and three of these IPT domains form PSI‐IPT domains with PSI domains.^7^, ^8^ Plexin C1 is an exception to this domain structure because it does not contain one of the PSI and two of the IPT domains.^10^ Plexin B1 (PLXNB1) also has a COOH‐terminal PSD‐95/Dlg/ZO‐1 (PDZ) binding motif.^11^ The PDZ binding motif of Plexin D1 (PLXND1) is essential for its ability to physically associate with the PDZ domain of GAIP‐interacting protein, C‐terminus (GIPC1).^12^ Among the 4 classes of PLXNs, PLXND1 is considered the most structurally diverse.^13^

The extracellular sema domain of Plexins is the distinctive structural and functional element of the semaphorin (SEMA) superfamily, which contains SEMAs, PLXNs and MET.^14^ Studies investigating Plexin A1 indicate that the interaction of the Sema domain with the proximal C‐terminal half of the ectodomain keeps Plexin inactive. After a plexin binds its ligand, the Plexin is activated.^15^ In addition, PLXNs mainly mediate the interaction with their SEMA ligands.^16^ This interaction is based on sema‐dependent dimerization and interaction specificity between PLXNs and SEMAs and transduces downstream signals to the cell through the cytoplasmic domain.^17^

SEMAs are extracellular signalling proteins that include both secreted (classes 2 and 3) and membrane‐tethered (classes 1, 4‐7 and V) forms.^18^ However, some members of the membrane‐tethered SEMA family, such as Semaphorin 4A (SEMA4A), Semaphorin 4D and Semaphorin 5A, can become soluble after proteolytic cleavage.^19^ Classes 1 and 2 are found in invertebrates, classes 3 to 7 are present in vertebrates, and class V SEMAs are found only in viruses.^20^ The functions of SEMAs are associated mainly with cytoskeletal control, including the control of cell migration, cell morphology and synapse remodelling.^21^ In the SEMA family, semaphorin 3A, semaphorin 3C (SEMA3C), semaphorin 3D (SEMA3D), semaphorin 3E (SEMA3E), SEMA4A and semaphorin 6D are ligands of PLXND1 in vertebrates.^18^ The canonical ligand of PLXND1 is SEMA3E.^12^

In addition, neuropilins (NRPs), which are single‐span transmembrane glycoproteins, serve as coreceptors for PLXNs and can be categorized into two classes, namely, NRP1 and NRP2.^22^ Typically, SEMAs bind either PLXN receptors or the PLXN‐NRP (NRP1/2) receptor complex to transduce their signals. While most membrane‐associated SEMAs directly bind PLXN receptors, most secreted SEMAs (except for SEMA 3E) require NRP1 or NRP2 to transmit their signals to PLXN receptors.^23^, ^24^

The cytoplasmic domain of Plexins is highly conserved, and their GAP activity leads to deactivation of R‐Ras, M‐Ras and Rap1, three small GTPases belonging to the Ras family.^25^ In the active state, R‐Ras GTPases enhance integrin‐mediated cell adhesion to the extracellular matrix (ECM).^26^ In a normal state, the Rap‐GAP activity of plexins is normally autoinhibited, and the dimerization of plexins can stimulate Rap‐GAP activity.^25^ Thus, upon ligand binding, PLXNs act as GAPs and modulate integrin‐mediated cell adhesion, cytoskeletal dynamics, and ERK and MAPK signalling.^27^ Notably, PLXNs are the only transmembrane receptors known to directly associate with small GTPases.^12^ Moreover, the RBD of PLXNs displays a ubiquitin‐like fold conformation.^28^ In PLXNB1, the RBD appears to be dimeric.^29^ However, in PLXND1, the RBD appears to be monomeric.^30^ In the inactive resting form, the cytoplasmic domain of plexins is dimerized via the RBD. Binding of a GTPase disrupts the dimer and induces conformational changes to convert the protein into an active state.^30^ In summary, concurrent binding of a SEMA to the extracellular domain and a Rho GTPase to the intracellular domain is required for the GAP activity of plexins. However, the molecular mechanisms responsible for the wide‐ranging effects of SEMAs and PLXNs are still far from clear.^31^

SEMA‐PLXND1 signalling plays an important role in cardiovascular, nervous, and immune system development and cancer biology.^13^, ^27^ PLXND1 expression is maintained during late embryonic development and early postnatal stages in the nervous system.^32^ SEMA3E‐PLXND1 signalling determines different types of synaptic specificity and formation depending on the ligand receptor localization on pre‐ and postsynaptic neurons.^33^ Recently, SEMA3E‐PLXND1 was confirmed to be a typical inhibitory cue for cortical and striatal neurons during brain development.^32^ The expression of PLXNs is often dysregulated in cancers.^34^ Some studies have shown that PLXND1 mediates protumorigenic signalling, while other studies indicate that PLXND1 facilitates antitumorigenic signalling.^13^ It seems that PLXND1 holds therapeutic importance as a biomarker in cancer and may also be a therapeutic target.

Recently, many studies have reported the emerging effects of PLXND1 in the cardiovascular system^27^, ^35^, ^36^, ^37^; particularly, a study by Ellie Tzima and colleagues described that PLXND1 functions as an endothelial mechanosensor and regulates vascular function and the site‐specific distribution of atherosclerosis.^35^ This finding suggests that a systematic summary of the emerging role of PLXND1 in cardiovascular development and disease, as well as the possible molecular mechanisms, is urgently needed. This understanding could help to elucidate the regulatory role of PLXND1 signalling in the cardiovascular system and may contribute to the identification of biomarkers and targets for cardiovascular disease diagnosis and treatment.

## 2 CARDIAC EFFECTS

### 2.1 Role of PLXND1 in cardiac development

**FIGURE 2:**
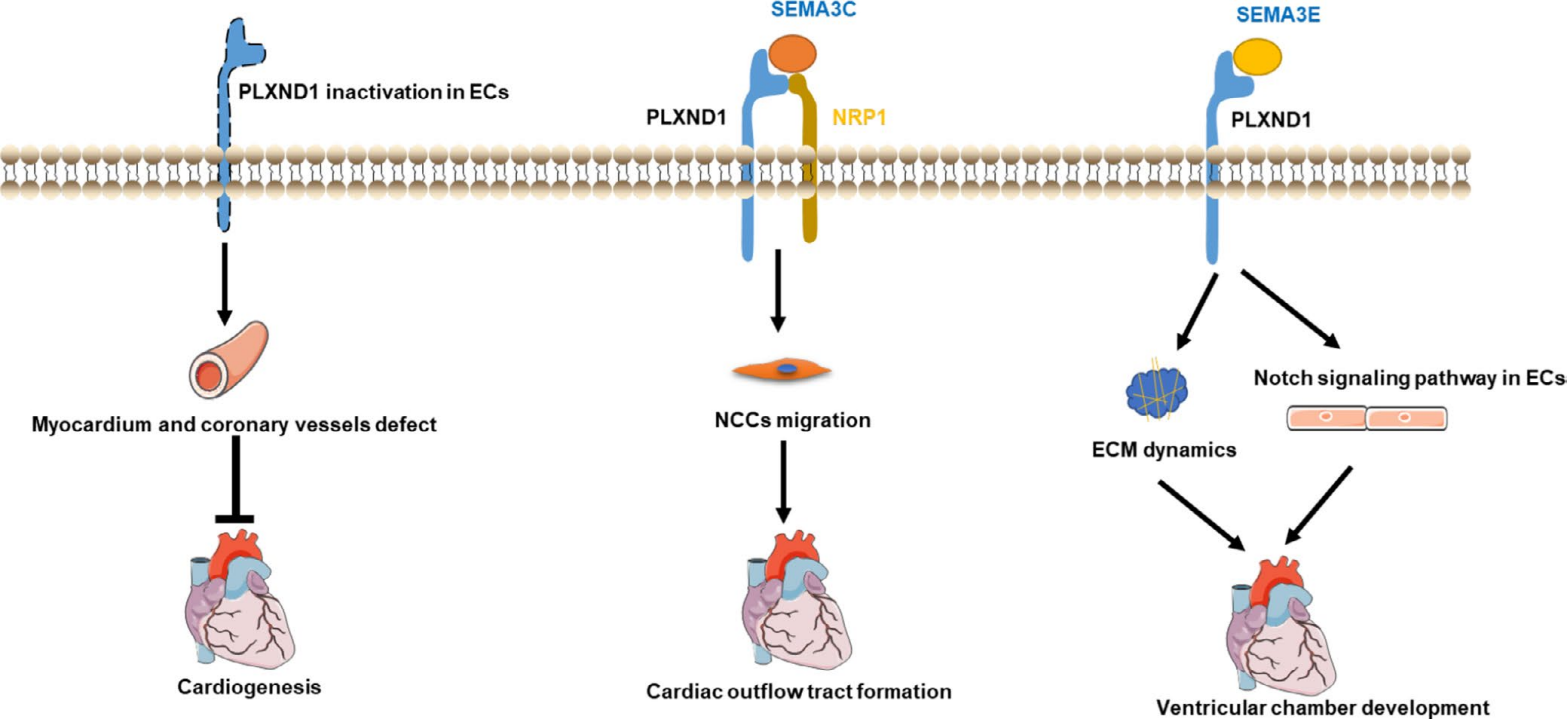
Regulatory roles of PLXND1 in cardiac development. PLXND1 is involved in the angiogenesis of the cardiogenesis; SEMA3C‐PLXND1/NRP1 signalling attracts neural crest cells (NCCs) to the cardiac outflow tract and plays a role in truncus arteriosus septation; SEMA3E‐PLXND1 signalling plays a critical role in ventricular chamber development by modulating extracellular cell matrix (ECM) dynamics and the Notch signalling pathway in ECs

PLXND1 plays an essential role in many aspects of heart development (Figure 2). PLXND1‐knockout mice die after birth and display cardiovascular abnormalities, including aortic arch anomalies, persistent truncus arteriosus (PTA), ventricular septal defects and decreased ventricular wall thickness.^38^ Additionally, PTA, ventricular septal defects, and coronary and atrial defects were detected in D1^ECKO^ mice, that is, mice with artificial PLXND1 mutation, which died of cyanosis after birth.^39^

Multiple cell types, including neural crest cells (NCCs), endothelial cells (ECs), myocardial cells, endocardial cells and epicardial cells, act coordinately during the complicated process of cardiogenesis in vertebrates.^18^ NCCs constitute a group of pluripotent cells that are generated at the edge of neural tubes and play a critical role in the separation of the aortic root and pulmonary trunk in truncus arteriosus.^40^ In fact, Semaphorin 6A and Semaphorin 6B act as contact repellents, driving NCCs towards the cardiac outflow tract (OFT) and initiating NCC migration from the dorsal neural tube through Plexin A2.^41^ Subsequently, SEMA3C attracts NCCs to the cardiac OFT through the PLXND1‐NRP1 receptor complex on the membrane of NCCs.^41^ Then, after migrating through the dorsolateral territory, cardiac NCCs reach their final target destinations and subsequently differentiate into endocardial and smooth muscle cells.^41^

The role of PLXND1 is not limited to the migration of cardiac NCCs. ECs constitute the endocardium, small blood vessels and coronary vessels in the heart.^42^ Research involving mice with endothelial compartment‐specific deletion of PLXND1 shows that inactivation of PLXND1 in ECs causes defects in the OFT independent of NCCs.^38^, ^39^ Both studies further confirmed that the absence of PLXND1 in ECs causes defects in the atrial muscle, ventricular wall and coronary vessels.^38^, ^39^ Additionally, endocardium‐myocardium communication is essential for trabeculation during cardiac development.^43^ Notch1 is expressed in endocardial cells near the trabecular myocardium, where it controls ventricular trabeculation and compaction by regulating Bmp10 signalling and Nrg1 expression.^44^ PLXND1 deletion elevates the expression of Notch pathway‐associated genes, including Notch1, its downstream effector Nrg1, its receptor ErbB2, Hrt1/2/3 and EphrinB2/B4, in the endocardium and leads to myocardial trabeculation and compaction defects.^37^ Considering these effects with similar phenotypes observed in mice with SEMA3E deletion, the authors suggest that SEMA3E‐PLXND1‐Notch signalling plays a critical role in ventricular chamber development.^37^ This finding suggests that PLXND1 activates endothelial functions to mediate appropriate cardiogenesis.

In addition to its functions in cells, PLXND1 regulates the abundance of the ECM between the endocardium and myocardium, which is essential for cardiac development.^37^ The ECM of the developing heart contains numerous molecules that play active and crucial roles between the endocardium and myocardium.^45^ Moreover, its degradation is essential for myocardial compaction.^45^ However, the ECM accumulates with myocardial compaction defects in the PLXND1^–/–^ heart.^37^ ECM proteolytic genes are also down‐regulated in the PLXND1^–/–^ heart.^37^ Considering these effects with similar phenotypes observed in mice with SEMA3E deletion, the authors suggest that SEMA3E‐PLXND1 signalling plays a critical role in ventricular chamber development by modulating ECM dynamics.^37^

### 2.2 Effects of PLXND1 on heart diseases

Numerous studies have demonstrated the essential role of PLXND1 and the corresponding SEMA ligands in animal heart development, but their role in human heart diseases has rarely been reported. Furthermore, information regarding the mechanisms and detection research investigating the effects of PLXND1 on human heart disease is lacking. However, the extremely important role of PLXND1 in animal heart development suggests that PLXND1 should be extensively studied in human heart diseases.

#### 2.2.1 Involvement of PLXND1 in congenital heart diseases (CHDs)

CHDs are found in 0.7% of live births and are a major cause of neonatal morbidity and mortality.^46^ PTA consists of a single arterial vessel overriding the two ventricles, which is the origin of the coronary, pulmonary and systemic arteries. PTA accounts for 0.9% of all cases of CHD (including live births, foetal deaths and terminated pregnancies).^47^ PTA may occur as an isolated malformation or a part of a genetic syndrome.^47^ Whole‐exome analysis of a DNA sample of a patient from a family with recurrent PTA revealed a missense mutation, Arg1299Cys (R1299C), in the PLXND1 gene.^47^ The role of PLXND1‐mediated signalling in PTA septation is further underscored by the occurrence of PTA in NRP1‐knockout animals, mutant animals with targeted disruption of SEMA3C, and patients and mice with GATA6 mutation.^47^ GATA6 directly regulates SEMA3C transcription, promoting its expression in the OFT.^48^ Thus, SEMA3C signalling propagated through the heterodimeric receptor PLXND1/NRP is important for TA septation. Moreover, Asaf and colleagues suggest that the R1229C mutation of PLXND1 is pathogenic in PTA.^47^

#### 2.2.2 Involvement of PLXND1 in CHARGE syndrome

CHARGE syndrome is a common cause of congenital anomalies affecting several tissues (Coloboma of the eye, Heart defects, Atresia of the choanae, Retardation of growth and/or development, Genital and/or urinary abnormalities, and Ear abnormalities and deafness) in a nonrandom fashion.^49^ All types of cardiac defects (except for heterotaxy and cardiomyopathy) have been documented, but conotruncal and atrioventricular septal defects are overrepresented.^50^ PLXND1‐knockout mice die quickly after birth.^38^ In contrast, SEMA3E‐knockout mice are viable and exhibit most but not all cardiovascular defects observed in PLXND1‐deficient animals.^24^, ^51^ Recently, a missense mutation in SEMA3E was identified in a patient suffering from CHARGE syndrome.^52^ Notably, CHARGE patients and SEMA3E‐ and PLXND1‐knockout mice show parallel abnormalities in the cardiovascular and nervous systems.^53^ Thus, Carl and colleagues suggested that impaired SEMA3E‐PLXND1 signalling was an aetiological factor in CHARGE syndrome.^12^

## 3 VASCULAR EFFECTS

### 3.1 Role of PLXND1 in the development of vessels

There are two main modes of vessel formation. In the developing mammalian embryo, angioblasts differentiate into ECs, which assemble into a vascular labyrinth via a process called vasculogenesis.^54^ The subsequent sprouting ensures the expansion of the vascular network, a process called angiogenesis. Then, arteriogenesis occurs, during which EC channels become covered by pericytes or vascular smooth muscle cells (VSMCs), which provide stability and control perfusion.^54^ ECs are common to all vessels and form the inner tunica intima layer that lines the vascular lumen. The tunica media of large elastic arteries and arterioles consist of VSMCs and elastic lamellae, and capillaries are covered by a discontinuous coat of pericytes instead of VSMCs. Compared to similar‐sized arteries, veins have a thinner media and are more compliant. Both arteries and veins have an outer tunica adventitia layer, which contains ECM, fibroblasts and progenitor cells.^55^ Patterning of the vascular system requires coordinated temporal and spatial direction for the developing endothelium and the ability of the endothelium to receive guidance signals.^56^ This coordination is accomplished by a combination of secreted attractive and repulsive factors, as well as cell‐to‐cell communication. Disruption of these signalling pathways can result in improper EC guidance, developmental pathologies and disease.^56^ Vascular endothelial growth factor (VEGF) is a highly specific endothelial growth factor. VEGF is thought to play an important role in physiological and pathological angiogenesis.^57^

PLXND1 is expressed in the vascular endothelium during embryogenesis in mice.^58^ The expression of PLXND1 is confined mainly to ECs associated with choroidal neovascularization and is evident in the ECs of normal retinal vessels in vivo.^59^ PLXND1 in zebrafish and mice reveals that SEMA selectively affects the cardiovascular system.^60^ Mutations in PLXND1 were identified in the zebrafish vascular patterning mutant out of bounds (obd).^61^ Mutants of PLXND1 exhibit vascular defects in intersegmental vessels (ISVs) positioning and patterning.^62^ In the dorsal skin of embryonic mice, genetic inactivation of PLXND1 results in abnormal artery‐lymph alignment and reduced lymphatic vascular branching.^63^ The PLXND1 signalling pathway seems to play an indispensable role in the development of embryonic blood vessels and postnatal angiogenesis. In fact, proper blood vessel pathfinding requires the endothelial receptor PLXND1 and SEMA signals.^61^

**FIGURE 3:**
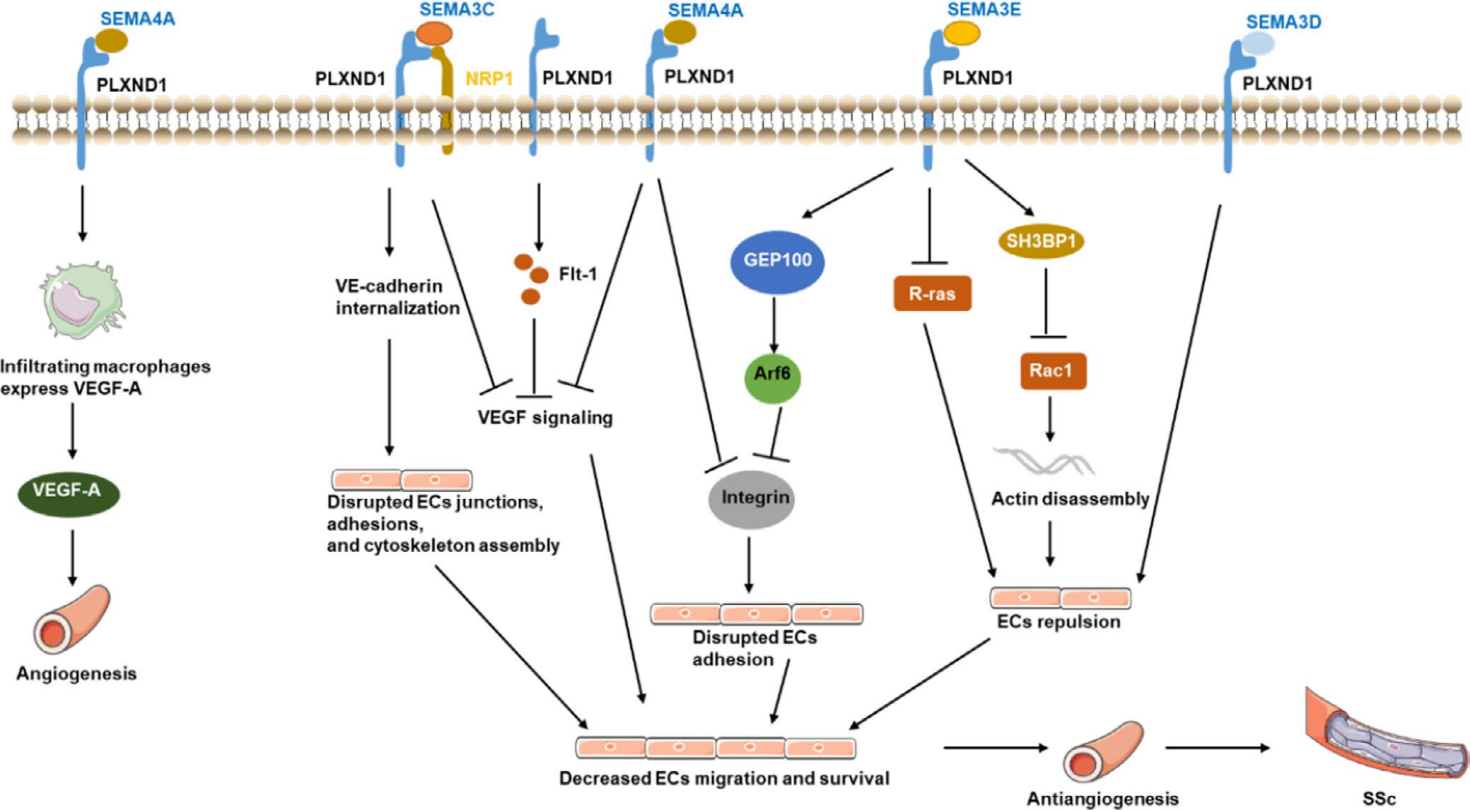
The effects of PLXND1 signalling in vessel development and systemic sclerosis (SSc). These signalling pathways mediate antiangiogenesis: SEMA3C‐PLXND1/NRP1 signalling induces VE‐cadherin internalization and suppress VEGF signalling, then disrupts endothelial cell (EC) junctions, focal adhesions and cytoskeleton assembly; PLXND1 signalling induces soluble flt‐1 expression to negatively control vascular endothelial growth factor (VEGF) signalling; SEMA4A‐PLXND1 signalling inhibits EC migration by suppressing the VEGF signalling and integrin‐dependent cell adhesion; SEMA3E‐PLXND1 signalling activates Arf6 by GEP100, resulting in the disassembly of integrin‐mediated focal adhesions; SEMA3E‐PLXND1 signalling deactivates the R‐ras, induces ECs repulsion; SH3BP1 mediates the inactivation of Rac1 induced by SEMA3E‐PLXND1 signalling to initiate actin disassembly; SEMA3D‐PLXND1 signalling mediates the repulsion of ECs; this signalling pathway mediates angiogenesis: SEMA4A‐PLXND1signalling could enhance the expression of vascular endothelial growth factor‐A in infiltrating macrophages, thereby activating angiogenesis

The zebrafish vasculature has high structural homology with that of other vertebrates, and most signalling pathways are highly conserved.^64^, ^65^ Zebrafish ISVs are an excellent system in which to study how alterations in signals result in subtle changes in cell morphology and behaviour, because zebrafish ISVs are only one cell wide and three or four cells long.^66^ ISVs are formed of angioblasts that sprout from the dorsal aorta, reach the dorsal region of the embryo and fuse with adjacent cells to form the dorsal longitudinal anastomotic vessel.^67^ A study showed that the loss of PLXND1 in obd mutants^67^ resulted in precocious ISV sprouting, while the loss of SEMA3E resulted in delayed ISV sprouting.^68^ SEMA3E thus seems to control the timing of ISV sprouting independent of PLXND1, indicating an antagonistic effect. The zebrafish common cardinal vein (CCV) can be used as a model of collective EC migration during cardiovascular morphogenesis.^69^ A study showed that SEMA3D is secreted from the mesenchyme and mediates the repulsion of ECs via the EC‐specific receptor PLXND1 in a paracrine manner in the CCV.^70^ During zebrafish segmental artery development, PLXND1‐mediated signalling limits the angiogenic potential of the aorta by inhibiting VEGF signalling via soluble flt‐1 (Figure 3). The specific member of the SEMA subfamily that mediates this event in zebrafish has yet to be identified.^71^ PLXND1 signalling induces soluble flt‐1 expression to negatively control VEGF signalling in zebrafish vessels.^16^

In addition to zebrafish‐related research, many studies on human cells have been conducted. Activation of PLXND1 by SEMA3E induces the interaction between the Ras GAP domain of PLXND1 and R‐Ras, thus sequestering R‐Ras and promoting rapid activation of ADP‐ribosylation factor 6 (Arf6), resulting in inactivation and subsequent internalization of integrins, thereby inhibiting EC adhesion to the ECM by disrupting integrin‐mediated adhesive structures and causing filopodial retraction in endothelial tip cells (Figure 3).^72^ Ultimately, this two‐pronged mechanism by which the SEMA3E‐PLXND1 signalling system acts may provide a repulsive signal to ECs, thereby protecting against the aberrant sprouting and growth of new blood vessels.^72^ Further research suggests that upon activation by SEMA3E, PLXND1 recruits phosphatidylinositol‐4‐phosphate 5‐kinase, and its enzymatic lipid product phosphatidylinositol 4,5‐bisphosphate binds the pleckstrin homology domain of GEP100.^73^ Binding of phosphatidylinositol 4,5‐bisphosphate to GEP100 enhances its guanine nucleotide exchange factor activity towards Arf6, resulting in the disassembly of integrin‐mediated focal adhesions and EC collapse.^73^ Another study demonstrated that SEMA3E‐PLXND1 down‐regulated Rac1 activity in human umbilical vein endothelial cells (HUVECs) and that this down‐regulation was mediated by SH3 domain‐binding protein 1 (SH3BP1), a RhoGAP protein.^74^ Moreover, upon SEMA3E binding, prebound SH3BP1 dissociates from the PLXND1 complex and converts Rac1‐GTP to Rac1‐GDP through its GAP activity to initiate actin disassembly. Finally, SH3BP1 is required for SEMA3E‐PLXND1‐mediated EC repulsion.^74^

More importantly, VEGF is necessary and sufficient to induce PLXND1 expression in angiogenic blood vessels. The SEMA3E‐PLXND1 signal is a negative feedback signal that modulates VEGF‐mediated angiogenic fate determination.^75^

SEMA3E is progressively reduced in VSMCs during vascular neointima formation. SEMA3E‐PLXND1 suppresses VSMC proliferation and migration via the inactivation of the Rap1‐AKT signalling pathways.^76^

Signalling by SEMA3E and its receptor PLXND1 controls EC positioning and the patterning of the developing vasculature in mice, and genetic ablation of SEMA3E or PLXND1 disrupts vascular patterning.^24^ SEMA3E signals through PLXND1 to regulate vascular patterning by modulating the cytoskeleton and focal adhesion structures.^77^

In addition to SEMA3E, a typical ligand of PLXND1, other ligands may play a role in vascular development by binding the PLXND1 receptor. SEMA4A inhibits EC migration and in vivo angiogenesis by suppressing VEGF‐mediated activation of Rac and integrin‐dependent cell adhesion (Figure 3). SEMA4A‐PLXND1 signalling appears to negatively regulate angiogenesis.^78^ Further research suggests that SEMA4A‐activated PLXND1 can also enhance the expression of VEGF‐A but not inflammatory chemokines in infiltrating macrophages, thereby activating angiogenesis.^79^ SEMA3C signals through the receptors NRP1 and PLXND1, which are strongly expressed on vascular tufts, induces VE‐cadherin internalization and abrogates VEGF‐induced activation of the kinases AKT, FAK and p38 MAPK. This signalling disrupts EC junctions, focal adhesions and cytoskeleton assembly, resulting in decreased cell migration and survival.^80^

In addition to those involved in the initial signalling pathway of PLXND1, other molecules may be involved in the regulation or downstream pathways of PLXND1. GIPC1 and its two paralogs GIPC2 and GIPC3 are universal adaptor proteins that bind and regulate the vesicular trafficking of many transmembrane proteins.^81^ Based on similar disturbed patterns of ISV organization in E11.5 GIPC1^−/−^ mouse embryos, mice lacking PLXND1, and PLXND1^+/−^ GIPC1^+/−^ heterozygous double mutant mouse embryos, Burk and colleagues proposed that PLXND1 and GIPC1 cooperate during vascular patterning.^82^ However, Jorge and colleagues drew the opposite conclusion, proposing that GIPC proteins negatively modulate PLXND1 signalling during vascular development in both zebrafish and mammals.^27^ These researchers found that zebrafish that endogenously express a PLXND1 receptor with predicted impairment of GIPC binding exhibited low‐penetrance angiogenesis deficits and antiangiogenic drug hypersensitivity.^27^ Moreover, GIPC mutant fish showed angiogenic impairments that were ameliorated by reducing PLXND1 signalling.^27^ Finally, GIPC depletion potentiated SEMA‐PLXND1 signalling in cultured ECs.^27^

In addition to its involvement in developmental angiogenesis, GIPC‐based modulation of endothelial PLXND1 signalling might promote the stabilization, repair, homeostasis and arteriogenic remodelling of vessels, particularly in environments with minimal proangiogenic stimulation. These conditions are found in the quiescent vascular beds of adults and are a hallmark of several diseases.^83^, ^84^

In summary, the role of PLXND1 signalling in vascular development has been elucidated by several in vivo and in vitro experiments (Figure 3). VEGF is involved in some signalling pathways, and GIPC proteins may negatively modulate PLXND1 signalling during vascular development.

### 3.2 Effects of PLXND1 on vessel diseases

#### 3.2.1 Involvement of PLXND1 in atherosclerosis

Atherosclerosis is a chronic inflammatory process initiated by EC dysfunction and the secretion of cytokines and chemokines.^85^ The ultimate outcome of atherosclerotic disease is characterized by plaque rupture and thrombus formation, which may lead to acute fatal coronary syndrome, myocardial infarction and stroke.^86^ During the early phase of atherosclerotic plaque formation, infiltrated monocytes differentiate into macrophages that take up oxLDL and other forms of modified LDL,^87^ subsequently forming foam cells that constitute fatty streaks.^88^ In addition, the migration and proliferation of VSMCs are crucial in neointimal formation, while neointimal hyperplasia is a pivotal pathophysiological process that contributes to atherosclerosis.^89^

**FIGURE 4:**
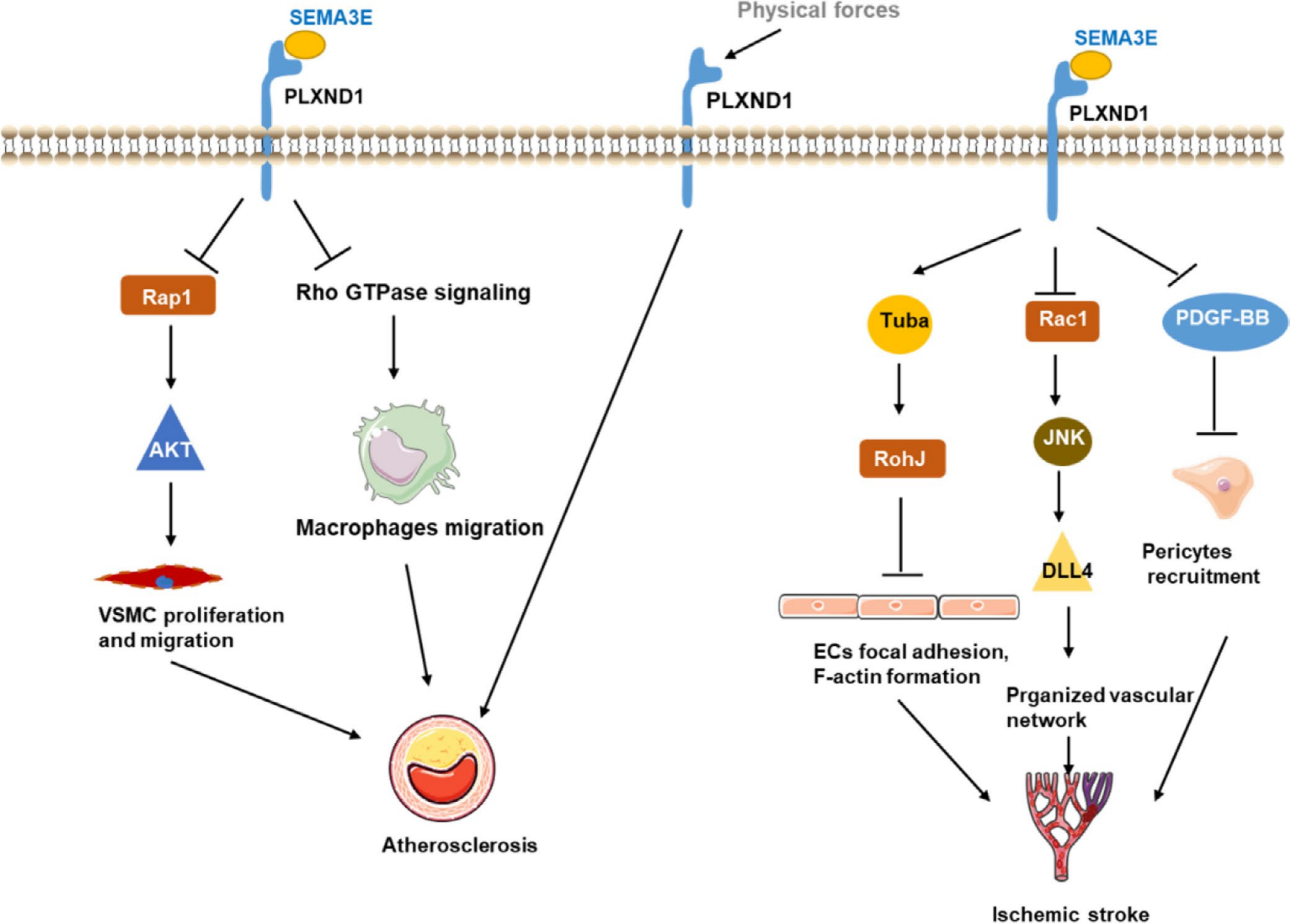
The roles of PLXND1 signalling during atherosclerosis and ischaemic stroke development. SEMA3E‐PLXND1 suppresses vascular smooth muscle cell (VSMC) proliferation and migration via the inactivation of the Rap1‐AKT signalling pathways; SEMA3E‐PLXND1 signalling inhibits the directional migration of macrophages by disrupting the Rho GTPase signalling cascade; PLXND1 is a direct force sensor during atherosclerosis; Ischaemic stroke: SEMA3E‐PLXND1 signalling inhibits the recruitment of pericytes by reducing platelet‐derived growth factor‐BB (PDGF‐BB) production in endothelial cells (ECs) after ischaemia; upon SEMA3E binding, Tuba dissociates from PLXND1 and increases RhoJ activity resulting in F‐actin disassembly and focal adhesion reduction; SEMA3E‐PLXND1 signalling inhibits DLL4 expression by inactivating the Rac1‐JNK signalling pathway under oxygen‐glucose deprivation and reoxygenation (OGDR) conditions

Recently, research has shown that SEMA3E and its receptor PLXND1 are highly expressed in macrophages in advanced atherosclerotic lesions in ApoE^–/–^ mice.^90^ SEMA3E‐PLXND1 signalling seems to inhibit the directional migration of macrophages by disrupting the Rho GTPase signalling cascade, the reorganization of the actin cytoskeleton and cell polarization in vitro (Figure 4).^90^ Another study showed that the levels of Sema3E and its receptor PLXND1 are markedly decreased in atherosclerotic plaques and VSMCs during neointimal hyperplasia.^76^ Mechanistically, SEMA3E, which plays a negative regulatory role in neointimal hyperplasia, inhibits VSMC migration and proliferation by inactivating the Rap1‐AKT signalling pathways by binding PLXND1 during neointimal formation.^76^, ^86^ Thus, during the early stage of atherosclerosis, neointimal hyperplasia is promoted by a reduction in signalling through the SEMA3E‐PLXND1 axis, while during the late stage of atherosclerosis, the directional migration of macrophages is inhibited by the SEMA3E‐PLXND1 signalling axis. Both processes are involved in the development of atherosclerosis.

Independent of SEMA3E, PLXND1 is a direct force sensor that forms a mechanocomplex with NRP1 and VEGFR2 that is necessary and sufficient for conferring mechanosensitivity upstream of the junctional complex and integrins on ECs.^35^ PLXND1 achieves its binary functions as either a ligand or a force receptor by adopting two distinct molecular conformations.^35^ Endothelial PLXND1 regulates signals at junctions and integrins and downstream cellular responses to shear stress in vitro and in vivo, ultimately regulating the site‐specific distribution of atherosclerosis (Figure 4).^35^

#### 3.2.2 Involvement of PLXND1 in systemic sclerosis (SSc)

SSc is an autoimmune connective disease in which ECs are dysfunctional with unregulated inflammation, leading to progressive fibrosis.^91^ SSc is associated with Raynaud's phenomenon (RP) and nailfold capillaroscopic changes.^92^ Collagen deposition, immune disorders and vascular abnormalities are currently proposed to be the three major causal factors of SSc.^93^ Vascular abnormalities usually develop during the initial phase of this disease and may exist in all phases.^94^ The earliest stage of SSc is characterized by morphologic alterations in vessel walls, such as capillary enlargement, intimal proliferation and fibrosis. EC injury plays a central role in promoting these changes, which are responsible for inducing hypoxia.^95^ In summary, EC injury and dysregulated angiogenesis seem to play central roles in the pathogenesis of SSc.^95^, ^96^, ^97^

A study showed that the serum levels of SEMA3E, a canonical ligand of PLXND1, in primary Raynaud's phenomenon (pRP) subjects and SSc patients were significantly higher than those in controls.^98^ This finding suggests that SEMA3E may lead to RP in SSc through the SEMA3E‐PLXND1 pathway. In addition, although the total expression of PLXND1 at the cell and tissue levels did not change significantly under different conditions, significantly higher levels of both the activated form of PLXND1 and SEMA3E were found in H‐MVECs challenged with sera from patients with SSc.^98^ PLXND1 not bound by SEMA3E also showed a significantly lower level in SSc‐MVECs and H‐MVECs challenged with sera from patients with SSc,^98^ possibly because SEMA3E does not stimulate PLXND1 expression and only activates existing PLXND1. Combined with those of the functional capillary experiment, these findings revealed that such activation of the SEMA3E‐PLXND1 pathway may contribute to defective angiogenesis during the early stage of SSc.^98^

Mechanistically, SEMA3E causes the interaction between PLXND1 and R‐Ras and the concomitant activation of Arf6, which interferes with the ability of integrin to bind the ECM, thus causing focal adhesion disassembly and EC detachment (Figure 3). The reduced adhesion of ECs to the ECM prevents their attachment and interferes with the growth of new sprouting vessels.^72^ Therefore, the SEMA3E‐PLXND1‐Arf6 signalling axis may initiate an antiangiogenic response during the early stage of SSc.

An in vitro experiment showed that SEMA4A binds PLXND1 in ECs, which mediates the antiangiogenic of these cells (Figure 3). The effects of SEMA4A on ECs depend on the suppression of VEGF‐mediated Rac activation and integrin‐dependent cell adhesion. SEMA4A‐PLXND1 signalling appears to negatively regulate angiogenesis.^78^ Furthermore, an in vitro experiment showed that PLXND1 receptors are responsible for transducing SEMA3C signals. SEMA3C inhibits sprouting angiogenesis by causing substantial rearrangement of the actin cytoskeleton, breakdown of adherens junctions and impairments in focal adhesion formation in HUVECs (Figure 3).^80^ SEMA3C inhibited VEGF‐mediated EC survival and cell migration and ultimately caused cell detachment and apoptosis in cellular models and in vivo.^80^ This finding suggests that SEMA3C‐PLXND1 signalling negatively regulates angiogenesis. Combined with the other inhibitory effects of PLXND1 on angiogenesis discussed above, these results indicate that signalling axes may play roles in SSc‐associated angiogenesis disorders (Figure 3).

#### 3.2.3 Involvement of PLXND1 in ischaemic stroke

Ischaemic stroke, a type of thrombo‐occlusive atherosclerotic disease, is a major cause of death and disability in the ageing population worldwide.^99^ Ischaemic stroke is the most common type of stroke.^100^ Angiogenesis is a crucial defence against hypoxia and regulates the process of long‐term neurologic recovery during the management of stroke.^36^ The model of vessel sprouting demonstrated that new branches in angiogenesis were spearheaded by tip cells.^101^ Only high expression of DLL4 in ECs determines tip cell selection.^102^ SEMA3E expression was up‐regulated in ischaemic tissue via a p53‐dependent pathway.^103^ Inhibition of SEMA3E‐PLXND1 signalling in the ischaemic penumbra, which increases both endothelial angiogenic capacity and the recruitment of pericytes, contributes to functional neovascularization and blood‐brain barrier integrity in aged rats.^104^ Mechanistically, SEMA3E‐PLXND1 signalling inhibits the recruitment of pericytes by reducing platelet‐derived growth factor‐BB (PDGF‐BB) production in ECs after transient middle cerebral artery occlusion (Figure 4).^104^ Upon SEMA3E treatment, Tuba dissociates from PLXND1 and converts RhoJ‐GDP to RhoJ‐GTP through its RhoGEF domain. The increased RhoJ activity leads to F‐actin disassembly and focal adhesion reduction.^36^ Moreover, SEMA3E‐PLXND1 inhibits DLL4 expression by inactivating the Rac1‐JNK signalling pathway under oxygen‐glucose deprivation and reoxygenation (OGDR) conditions.^36^ This finding suggests that inhibiting SEMA3E‐PLXND1 signalling is a novel therapeutic strategy for improving brain tissue survival and functional recovery after ischaemic stroke.

Inhibition of SEMA3E‐PLXND1 signalling is also a therapeutic strategy under other ischaemic conditions. It has been reported that hyperglycaemia activates p53 by increasing the production of reactive oxygen species.^105^ Thus, p53‐induced up‐regulation of antiangiogenic factors (including SEMA3E) likely accounts for the impairment of angiogenesis in patients with diabetes.^103^ Therefore, SEMA3E‐PLXND1 could also be a target for the treatment of ischaemic cardiovascular disease in diabetic patients, because conventional therapeutic angiogenesis is not very efficient in this patient population.^103^ Notably, in a mouse model of ischaemic retinopathy, high expression of endothelial PLXND1 and RhoJ was limited to abnormal extraretinal vessels (Figure 4). By targeting PLXND1, intravitreal injection of the SEMA3E protein selectively suppressed the disorganized outgrowth of extraretinal vessels, leading to the subsequent regeneration of normal vasculature in ischaemic retinas.^106^

## 4 CONCLUSIONS AND PERSPECTIVE

**FIGURE 5:**
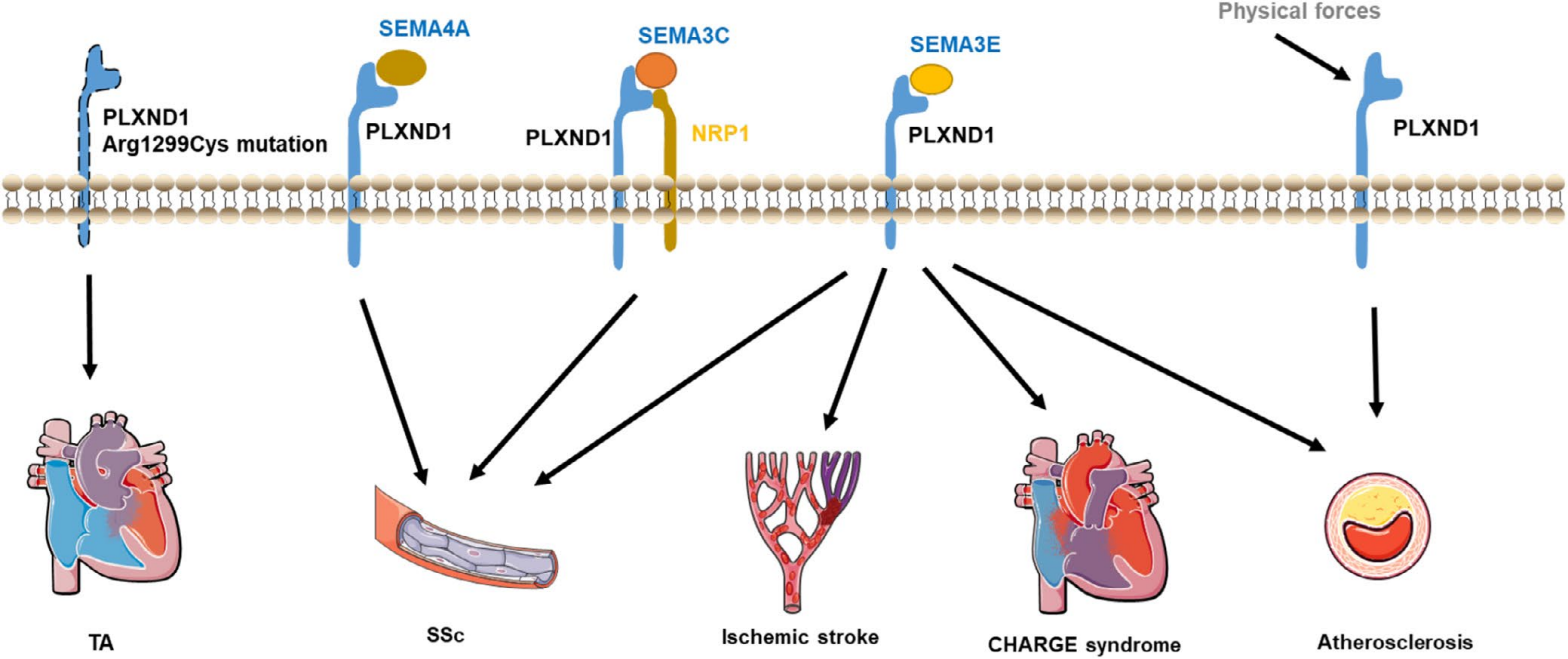
Different roles of PLXND1 signalling in various cardiovascular diseases. PLXND1 Arg1299Cys mutation was revealed in a truncus arteriosus (TA) patient; SEMA4A‐PLXND1, SEMA3E‐PLXND1 and SEMA3C‐PLXND1/NRP1 signalling may play roles in SSc‐associated angiogenesis disorders; SEMA3E‐PLXND1 signalling may play roles in SSc, CHARGE syndrome and atherosclerosis and may inhibit angiogenesis after ischaemic stroke; PLXND1 is a direct force sensor during atherosclerosis

Accumulating evidence indicates that PLXND1 has distinct biological effects, which are not limited to the nervous system but also include other systems and physiological and pathophysiological processes, including cancer, the immune system and especially the cardiovascular system. Work conducted over the past decade has led to the identification of many new interactions between PLXND1 and specific SEMAs. To date, the function of PLXND1 in the cardiovascular system has not been fully elucidated. In this review, we discuss the regulatory roles of PLXND1 signalling in heart and vessel development and various cardiovascular diseases (Figure 5).

PLXND1‐knockout mice die after birth and exhibit cardiovascular abnormalities. Possibly because of the high lethality of PLXND1 deletion, most human PLXND1 mutations may cause severe fatal congenital diseases that cannot be reported. The missense mutation Arg1299Cys in the PLXND1 gene was identified in a patient from a family with recurrent TA, a CHD. The essential role of PLXND1 in cardiac OFT formation and myocardial trabeculation has been reported. PLXND1 also activates the endothelial functions necessary for correct cardiogenesis.

The zebrafish is often used as a model in vascular studies because of the high structural homology between its vasculature and that of other vertebrates and its highly conserved signalling pathways. The role of PLXND1 in blood vessels has also been studied in many human cells. PLXND1 mutation or deletion can lead to angiogenesis disorders and patterning error defects. PLXND1 appears to have an inhibitory effect on angiogenesis, which may protect normal vascular structures from pathological angiogenesis. PLXND1 is often activated by SEMAs (primarily SEMA3E) and is involved in physiological and pathophysiological processes in blood vessels. There is also a mutual connection between PLXND1 and VEGF. In particular, PLXND1, which acts as a mechanical force sensor, can regulate the site‐specific distribution of atherosclerosis. Inhibition of SEMA3E‐PLXND1 signalling has been suggested as a therapeutic strategy for ischaemic conditions.

Despite these important findings, our understanding of PLXND1 biology is still limited, and many questions and areas for future research remain. These areas include the following. (1) To date, the cardiac effects of PLXND1 signalling have been poorly studied. The different roles of PLXND1 during the different stages of cardiac development and morphology can be further elucidated. (2) The proteins in downstream signalling cascades activated after ligand binding to PLXND1 in which PLXND1 acts as a mechanical force sensor, such as RhoGAP proteins and, the PLXND1 complex, are far from clear. (3) How PLXND1 signalling interacts with other signalling pathways, such as the VEGF‐VEGFR signalling pathways and the Notch signalling pathway, remains an open question.

It can be concluded that the answers to these questions could provide the scientific community insight into the role of PLXND1 in the cardiovascular system and most likely yield new biomarker(s) and ultimately help to identify new therapeutic target(s) for cardiovascular diseases.

## CONFLICTS OF INTEREST

The authors declare that they have no conflict of interest.

## AUTHOR CONTRIBUTION

**Yi‐Fei Zhang:** Writing‐original draft (lead). **Yu Zhang:** Formal analysis (equal). **Dong‐Dong Jia:** Formal analysis (equal). **Hong‐Yu Yang:** Methodology (equal). **Mengdie Cheng:** Methodology (equal). **Wen‐Xiu Zhu:** Writing‐review & editing (supporting). **Hui Xin:** Writing‐review & editing (supporting). **Pei‐feng Li:** Writing‐review & editing (supporting). **Yin‐Feng Zhang:** Conceptualization (lead); Writing‐original draft (supporting); Writing‐review & editing (lead).

## Data Availability

Research data are not shared in this article, because no new data were created or analysed in this study.
